# Integrated identification of key genes and pathways in Alzheimer’s disease via comprehensive bioinformatical analyses

**DOI:** 10.1186/s41065-019-0101-0

**Published:** 2019-07-16

**Authors:** Tingting Yan, Feng Ding, Yan Zhao

**Affiliations:** Department of Bioengineering, Harbin Institute of Technology, Weihai, 264209 Shandong China

**Keywords:** Alzheimer’s disease, Differently expressed genes, Systematic biological analyses, Key candidate genes, lncRNAs

## Abstract

**Background:**

Alzheimer’s disease (AD) is known to be caused by multiple factors, meanwhile the pathogenic mechanism and development of AD associate closely with genetic factors. Existing understanding of the molecular mechanisms underlying AD remains incomplete.

**Methods:**

Gene expression data (GSE48350) derived from post-modern brain was extracted from the Gene Expression Omnibus (GEO) database. The differentially expressed genes (DEGs) were derived from hippocampus and entorhinal cortex regions between AD patients and healthy controls and detected via Morpheus. Functional enrichment analyses, including Gene Ontology (GO) and pathway analyses of DEGs, were performed via Cytoscape and followed by the construction of protein-protein interaction (PPI) network. Hub proteins were screened using the criteria: nodes degree≥10 (for hippocampus tissues) and ≥ 8 (for entorhinal cortex tissues). Molecular Complex Detection (MCODE) was used to filtrate the important clusters. University of California Santa Cruz (UCSC) and the database of RNA-binding protein specificities (RBPDB) were employed to identify the RNA-binding proteins of the long non-coding RNA (lncRNA).

**Results:**

251 & 74 genes were identified as DEGs, which consisted of 56 & 16 up-regulated genes and 195 & 58 down-regulated genes in hippocampus and entorhinal cortex, respectively. Biological analyses demonstrated that the biological processes and pathways related to memory, transmembrane transport, synaptic transmission, neuron survival, drug metabolism, ion homeostasis and signal transduction were enriched in these genes. 11 genes were identified as hub genes in hippocampus and entorhinal cortex, and 3 hub genes were identified as the novel candidates involved in the pathology of AD. Furthermore, 3 lncRNAs were filtrated, whose binding proteins were closely associated with AD.

**Conclusions:**

Through GO enrichment analyses, pathway analyses and PPI analyses, we showed a comprehensive interpretation of the pathogenesis of AD at a systematic biology level, and 3 novel candidate genes and 3 lncRNAs were identified as novel and potential candidates participating in the pathology of AD. The results of this study could supply integrated insights for understanding the pathogenic mechanism underlying AD and potential novel therapeutic targets.

**Electronic supplementary material:**

The online version of this article (10.1186/s41065-019-0101-0) contains supplementary material, which is available to authorized users.

## Background

Alzheimer’s disease (AD) is recognized as the most common neurodegenerative disease and a typical hippocampal amnesia, and also one of the dominating deadly disease affecting elderly population. The disease is characterized by the extracellular senile plaques formed by amyloid-β (Aβ) peptides, intracellular neurofibrillary tangles (NFTs), and also structure and function changes of brain regions related to memory [1–3]. It is well known that AD has complex multiple pathogenic factors, such as genetic factor, environmental factor, immunological factor, head injuries, depression, or hypertension [4–8]. Among these factors, genetic factors are estimated to attribute about 70% to the risk for AD [9]. Dominant mutations of genes encoding APP (amyloid precursor protein), PSEN1 (presenilin 1), and PSEN2 (presenilin 2), which enhanced generation and aggregation of Aβ, were included in the established genetic causes of AD [10]. However, APP, PSEN1 and PSEN2 are only partially accountable for the pathogenic mechanism of AD patients [11, 12]. Besides, genetic analyses have demonstrated that, individual differences of AD could be resulted from multiple genes and their variants, which exert various biological functions in coordination to enhance the risk of the disease [13–15]. Except for identifying mechanisms involved in the AD pathogenesis, comprehensive analyses of potential candidate genes could suggest novel potential strategies to predictive or diagnostic test for AD.

Hub genes, regulatory transcription factors and microRNAs in the entorhinal cortex tissues of mid-stage AD cases have been identified via analyzing the database of GSE4757 and therapeutic targets or biomarkers of the AD were demonstrated in previous study [16]. Multiple methods were employed in the identification of potential molecules targets and drug candidates to AD, and hub genes like ZFHX3, ErbB2, ErbB4, OCT3, MIF, CDK13, GPI and so forth were found in the analyses of current datasets, such as GSE48350, GSE36980, GSE5281, and so forth [17–23]. Whereas, the remarkable and integrated details of key candidate genes and pathways related with the pathogenesis of AD are still incomplete. Furthermore, it is well documented that long non-coding RNAs (lncRNAs) play vital roles in the regulation of gene expression epigenetic, transcriptional, and posttranscriptional levels [24–26], and only several lncRNAs have been validated to be involved in the pathogenesis of AD [27–30]. Given that sufficiently illuminating human lncRNA-AD associations have great potential benefit to diagnosis, prevention, treatment, and prognosis of AD, it is an urgent task to find novel connections between lncRNA and AD.

In the present study, we implemented integrated analyses of genes involved in AD from the information filtration of the database, GSE48350 [31–33]. We employed Morpheus, an online tool, to identified differentially expressed genes (DEGs). Then biological enrichment analyses were conducted to detect the remarkable functional terms and analyzed the reciprocities among the biological pathways enriched by pathway analyses methods. Moreover, a protein network specific in AD was speculated and evaluated in the background of the human protein-protein interaction (PPI) network. 3 novel genes and 3 lncRNAs were differently expressed in the tissues of hippocampus and/or entorhinal cortex between AD patients and normal ones were identified as novel and potential candidates of AD pathology, and binding proteins of these lncRNAs closely associate with pathogenic mechanism of AD. The results of the present study should supply ponderable hints for understanding the pathogenesis molecular mechanisms of AD from a standpoint of systems biology.

## Results

### Identification of DEGs

The gene expression profile and sample information of post-mortem brain tissue samples of AD patients and normal people of GSE48350 were obtained from National Center of Biotechnology Information-Gene Expression Omnibus (NCBI-GEO) and ArrayExpress database, respectively, which are free databases of microarray/gene profile and next-generation sequencing. There were total 253 samples in this database, including microarray data from normal controls and AD cases aged 20–99 years, from 4 brain regions: hippocampus, entorhinal cortex, superior frontal cortex, and post-central gyrus. We analyzed the differences of gene expression between 18 AD samples (69–99 years old) and age-matched 24 normal samples of hippocampus tissues, and 15 AD samples (69–99 years old) and age-matched 17 normal samples of entorhinal cortex tissues in the present study (Additional file 1: Table S1). Employing Morpheus software and using *p* < 0.05 and |log2FC| ≥ 1 (FC, fold change) as cut-off criterion, 251 genes (56 up-regulated and 195 down-regulated genes) and 74 genes (16 up-regulated and 58 down-regulated genes) were identified as DEGs in the AD samples compared with the normal ones in the tissues of hippocampus and entorhinal cortex, respectively (Table 1 and Additional file 2: Table S2).

**Table 1:** DEGs were identified from the dataset

	Gene symbol
Up-regulated genes (hippocampus tissues)	ANKIB1 SLC25A46 ZNF621 XIST C1orf87 MAP 3 K19 CD163 LTF AGBL2 PZP TAC1 IGFBP7-AS1 SLC27A6 C1orf192 CD44 SYNE2 FAM216B ABCA6 SPATA18 CCDC11 FAM81B SERPINI2 CRLF1 WDR49 TNFRSF11B DNAAF3 CDK19 RASSF9 FREM3 ANKFN1 ZBBX ART3 CAPS SLC7A11 PIH1D3 WWTR1 CCDC81 EFCAB1 TEX26 EFEMP1 DNAH6 SLC19A3 C21orf62 TDGF1 /// TDGF1P3 MORN5 STON2 CP DYNC2H1 ECM2 WDR96 CXorf30 FANCB CDC14A LEPR /// LEPROT FHL5 ARHGEF7
Down-regulated genes (hippocampus tissues)	NWD2 ADAD2 BSN AMPH SLC26A10 NRP1 CYP2A7P1 EBP PCLO ETNK1 SLC24A3 KIT KCNJ6 DDX50 CD300C DIRAS3 RAB3C TTC5 KCNG3 KALRN SCNN1G ACSL4 MICAL2 MAP 7D2 CALY SOX1 C16orf74 DNASE1L2 NEFH ANK1 COL2A1 LOC100132891 RCOR3 GABRG2 SPDEF ACHE RDH10 TRAF3IP1 CNR1 TMEM35 PDPN F12 KCNC1 OBSCN STC2 EXOSC3 ACTRT3 RHCG DGAT2 SLC35G1 CAMK1 KCNK10 DCAF15 METTL10 GLS2 FHL2 MICB MCHR1 PPM1E TP53I11 KCMF1 CELF4 SCG2 SCN2B VAPA CARTPT NRP2 CDC42 MIR22 /// MIR22HG BRSK2 SPANXA1 /// SPANXA2 /// SPANXB1 /// SPANXC ATP1A3 SLC2A3 PLK2 HOMER2 GFOD1 NCDN GLRX PCOLCE2 MATN1 NFKBID LAMP5 LY86-AS1 RPL27A /// SNORA45A SYN2 KCNA1 KCNJ5 GSDMB SLC6A3 CADPS ATP8A2 SRGAP3 NUP93 TMEM155 QRICH1 IL4I1 DHRS2 AFF2 KISS1R STRIP2 TRPV2 SGPP2 OPRK1 SLC22A8 SSTR2 SLC1A6 C14orf180 RIMKLA KLK7 RET NKX2–5 ACTN1 SPAG11A LEPREL2 IGFBPL1 FABP3 MAGEL2 PPP1R17 IL12RB1 SYT2 SNORD114–3 AFF3 PCDH20 LRRFIP1 LINC00622 L3MBTL1 PYCRL TBC1D26 /// ZNF286A ATF7IP2 PWP2 CACNA1A KCNJ4 TRHDE SUSD4 UBASH3B SIAH1 PTH2R SDK1 CCNJL KCNQ5 TMEM61 PML NRIP3 METTL7B LINC00282 WIPF3 CLSTN2 GREM2 LOC389906 IFRD1 MRAP2 CENPVP1 /// CENPVP2 KCNIP2 KDM6B CTSG KMT2D CPNE4 PGM5 CARD14 CST7 SPOCD1 HINT3 NR4A3 CENPW VEGFA PCDH8 PKIB DNAJB5 ARC GRP CKS2 PTGS1 ARHGAP36 A1BG RTF1 AQP3 TSPAN18 TH CHGB CITED1 CALB1 CRABP1 MIR7-3HG PDYN FNDC9 SCGN WFIKKN2 BDNF RAE1 INHBA EGR4 FOSB LAMB3 EGR2 MPO NPAS4
Up-regulated genes (entorhinal cortex tissues)	ANKIB1 TAC1 SLC25A46 XIST ZNF621 C1orf192 SPATA13 PKP2 CDK19 A2ML1 CX3CR1 C1orf87 RAPGEF5 CD24 ID2 /// ID2B DST
Down-regulated genes (entorhinal cortex tissues)	RIT2 SLC17A6 POPDC3 RPLP2P1 /// RPLP2P1 NEFH CKS2 LRRFIP1 CYP27C1 CABP1 SCGB3A1 LY86-AS1 VASH2 QPCT CENPF MPP7 TWIST2 COL4A1 TCERG1L RGS2 HSPB3 ANKRD34C OTP KDELR3 TPSAB1 BRE-AS1 CARTPT SECTM1 XIRP1 IL1RL1 GPR68 MMP10 TPSB2 DUSP2 RSPO2 PTGS2 RTF1 IL7R GPR3 FOSL1 LINC00622 IL11 DHRS2 FOSB CBLN1 SAA1 /// SAA2 C2CD4A SPOCD1 TGFBI HDC HS3ST2 LINC00960 ATP1B4 RAE1 PMCH LAMB3 OXT INHBA AVP

56 up-regulated genes and 195 down-regulated genes were included in the hippocampus tissues of AD patients’ samples compared to normal hippocampus tissues; meanwhile, 16 up-regulated genes and 58 down-regulated genes were included in the entorhinal cortex tissues of AD patients’ samples compared to normal entorhinal cortex tissues. (The up-regulated genes were listed from the largest to the smallest of fold changes, and down-regulated genes were listed from the smallest to largest of fold changes)

### Gene ontology (GO) enrichment analyses of DEGs

**Fig. 1:**
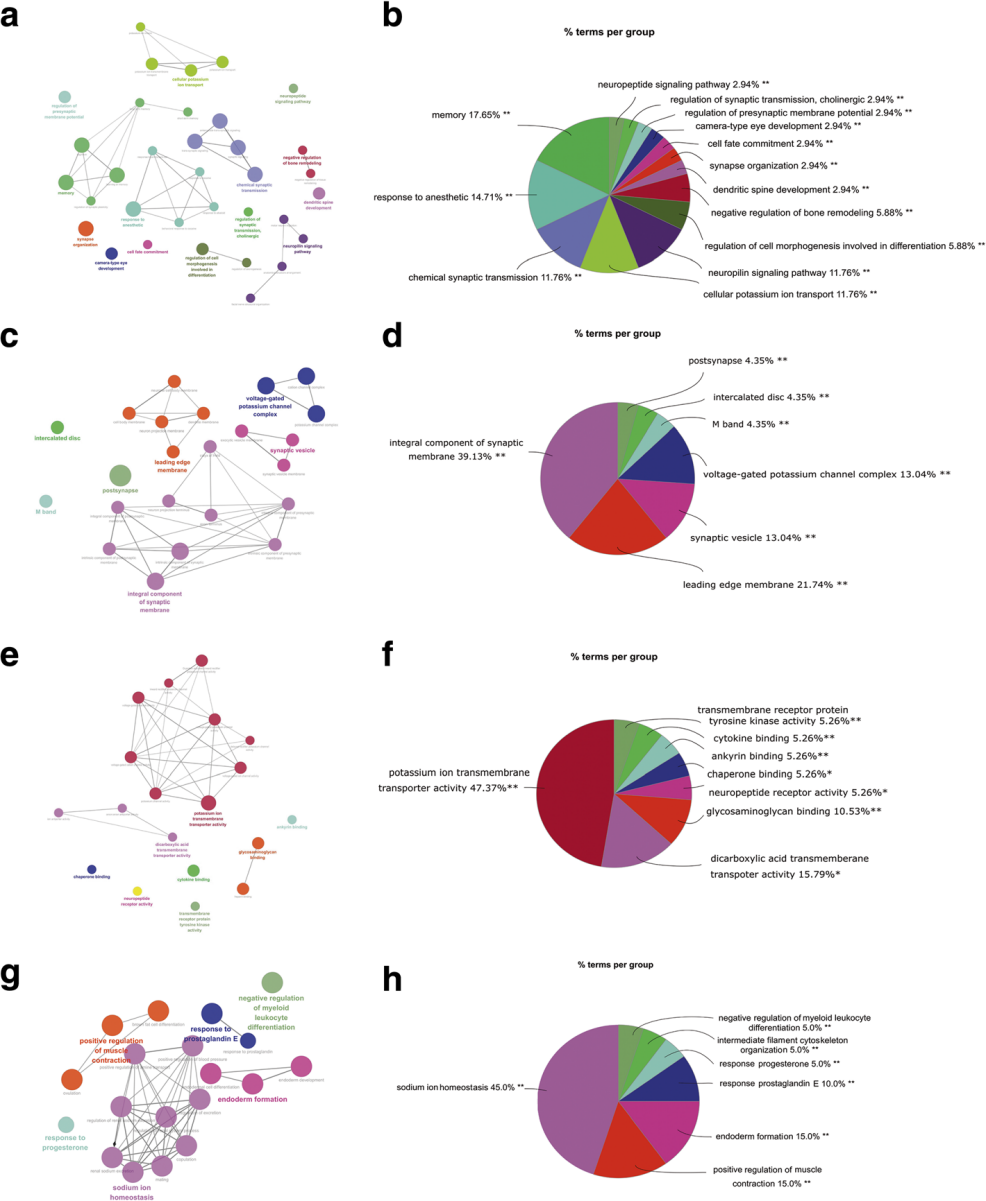
GO analyses of DEGs. 86 DEGs from hippocampus tissues were mapped to 34 different biological processes (a)(b). **a** Group information of biological processes. **b** Percentages of biological processes terms per group. 54 DEGs from hippocampus tissues were mapped to 23 different cellular components (c)(d). **c** Group information of cellular components. **d** Percentages of cellular components terms per group. 44 DEGs from hippocampus tissues were mapped to 19 different molecular functions (e)(f). **e** Group information of molecular functions. **f** Percentages of molecular functions terms per group. 17 DEGs from entorhinal cortex tissues were mapped to 20 different biological processes (g)(h). **g** Group information of biological processes. **h** Percentages of biological processes terms per group

GO analyses for the DEGs after gene integration were performed via Cytoscape and its plugs, Cluego and Cluepedia. 86 of the 251 DEGs from hippocampus tissues were mapped to 34 different biological processes (Fig. 1a), of which prominent examples are memory 17.65%, response to anesthetic 14.71%, chemical synaptic transmission 11.76% and cellular potassium ion transport 11.76% and neuropilin signaling pathway 11.76% (Fig. 1b and Additional file 3: Table S3). 54 of the 251 DEGs from hippocampus tissues were mapped to 23 different cellular components (Fig. 1c), of which prominent examples are integral component of synaptic membrane 39.13%, leading edge membrane 21.74% (Fig. 1d and Additional file 4: Table S4). 44 of the 251 DEGs from hippocampus tissues were mapped to 19 different molecular functions (Fig. 1e), of which prominent examples are potassium ion transmembrane transporter activity 47.37%, dicarboxylic acid transmembrane transporter activity 15.79% (Fig. 1f and Additional file 5: Table S5). 17 of the 74 DEGs from entorhinal cortex tissues were mapped to 20 different biological processes (Fig. 1g), of which prominent examples are sodium ion homeostasis 45%, positive regulation of muscle contraction 15% and endoderm formation 15% (Fig. 1h and Additional file 6: Table S6).

### Pathway enrichment analyses of DEGs

In all, pathway enrichment analyses of DEGs were classified by the Kyoto Encyclopedia of Genes and Genomes (KEGG), Reactome and Wikipathway databases, respectively, using *p* < 0.05 as cut-off value. The different databases provided similar information with the majority of AD-related proteins acting in 19 major pathways (13 from hippocampus and 6 from entorhinal cortex), mainly related transmembrane transportation, drug reactions, synapses function, ion homeostasis, neurogenesis and signal transduction (Additional file 7: Table S7).

### PPI network analyses and module analyses

**Fig. 2:**
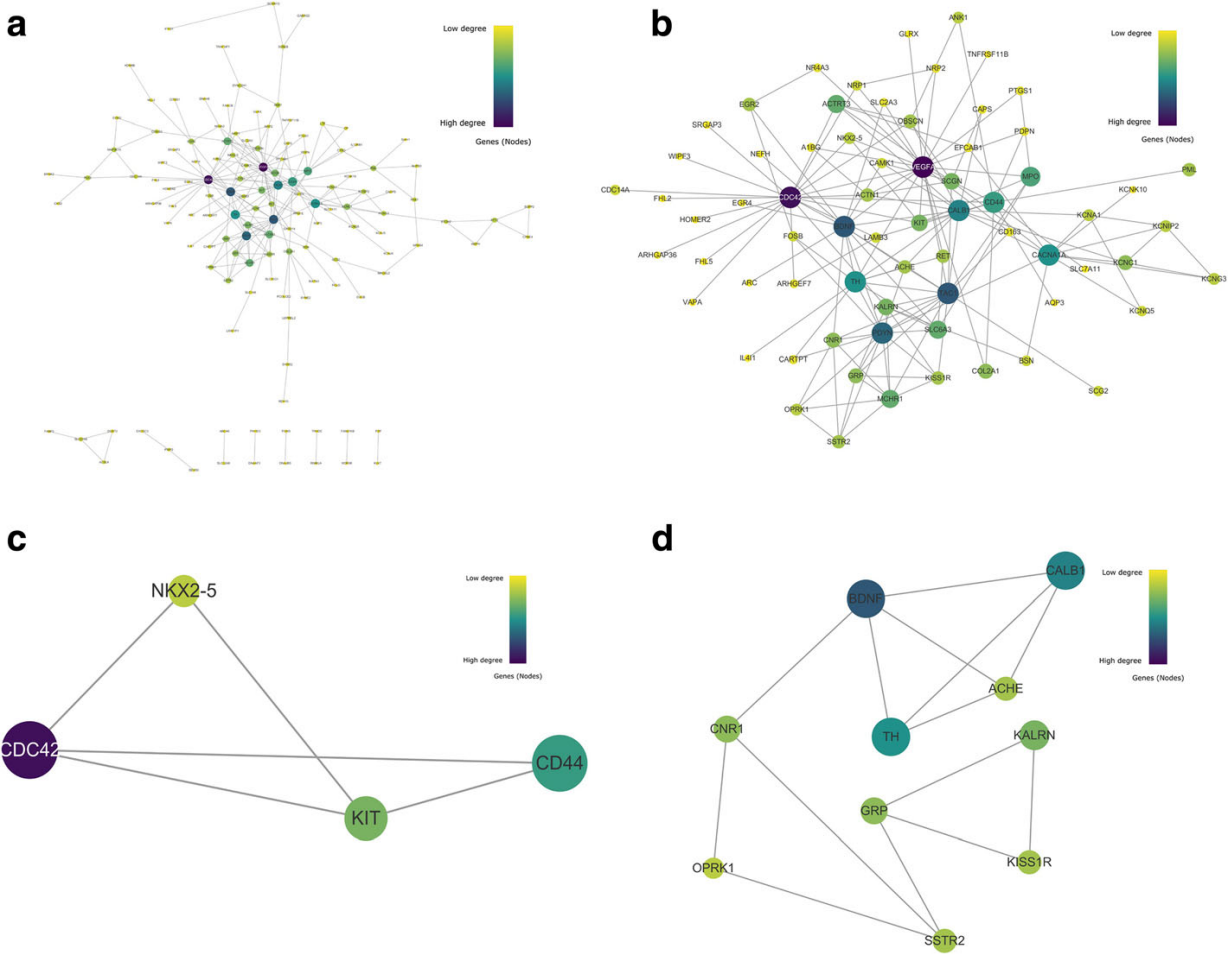
PPI networks and clusters of DEGs from hippocampus. **a** PPI networks of all 135 proteins. **b** Hub genes and their neighbor genes. **c** Module 1 from the PPI network. **d** Module 2 from the PPI network

Using the Search Tool for the Retrieval of Interacting Genes database (STRING) online database and Cytoscape software, total of 135 DEGs (26 up-regulated and 109 down-regulated genes) of the 251 commonly altered DEGs from hippocampus were screened into the DEGs PPI network, containing 135 nodes and 221 edges (Fig. 2a), and 116 of the 251 DEGs did not fall into the DEGs PPI network. Based on the STRING database, we made the PPI network of a total of 135 nodes and 221 protein pairs was obtained with a combined score > 0.4. As shown in Fig. 2a, the majority of the nodes in the network were down-regulated DEGs in AD samples. Among the 135 nodes, 9 central node genes were identified with the filtering of degree≥10 criteria (i.e., each node had more than 10 connections/interactions) as top 9 hub genes, which were CDC42, BDNF, TH, PDYN, VEGFA, CALB, CD44, TAC1 and CACNA1A (Fig. 2b). Figure 2b presents these 9 genes and their first neighbor genes.

In total, 2 modules (Modules 1 and 2) with score > 3 were detected by Cytoscape plug-in Molecular Complex Detection (MCODE) (Fig. 2c and d). KEGG pathway enrichment analyses showed that Module 1 consisted of 4 nodes and 5 edges, which are mainly associated with Endocytosis, Rap1 signaling pathway and Ras signaling pathway (Table 2), and that Module 2 consisted of 10 nodes and 14 edges, which are mainly associated with neuroactive ligand-receptor interaction, and cocaine addiction (Table 3).

**Table 2:** Pathway enrichment in Module 1

#Pathway ID	Pathway description	Observed gene count	False discovery rate	Matching proteins in your network (ids)	Matching proteins in your network (labels)
5131	Shigellosis	2	0.0131	ENSP00000314458, ENSP00000398632	CD44,CDC42
4640	Hematopoietic cell lineage	2	0.0146	ENSP00000288135, ENSP00000398632	CD44,KIT
4014	Ras signaling pathway	2	0.0323	ENSP00000288135, ENSP00000314458	CDC42,KIT
4015	Rap1 signaling pathway	2	0.0323	ENSP00000288135, ENSP00000314458	CDC42,KIT
4144	Endocytosis	2	0.0323	ENSP00000288135, ENSP00000314458	CDC42,KIT
5205	Proteoglycans in cancer	2	0.0323	ENSP00000314458, ENSP00000398632	CD44,CDC42

**Table 3:** Pathway enrichment in Module 2

#Pathway ID	Pathway description	Observed gene count	False discovery rate	Matching proteins in Your network (ids)	Matching Proteins In your Network (labels)
4080	Neuroactive ligand- receptor interaction	4	0.00166	ENSP00000234371, ENSP00000265572, ENSP00000350198, ENSP00000358511	CNR1, KISS1R, OPRK1, SSTR2
5030	Cocaine addiction	2	0.0359	ENSP00000370571, ENSP00000414303	BDNF,TH

**Fig. 3:**
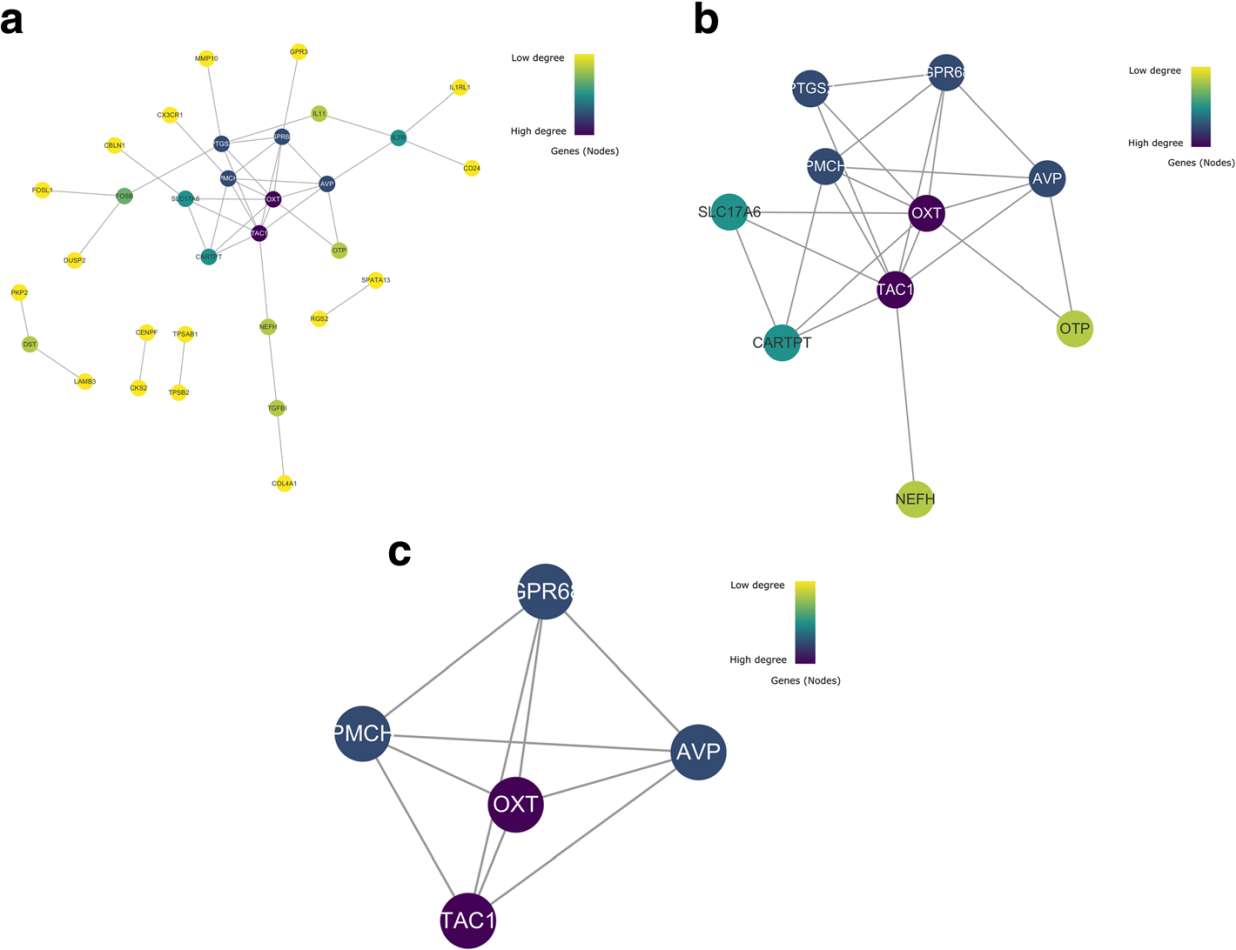
PPI networks and clusters of DEGs from entorhinal cortex. **a** PPI networks of all 32 proteins. **b** Hub genes and their neighbor genes. **c** Module 3 from the PPI network

Similarly, total of 32 DEGs (6 up-regulated and 26 down-regulated genes) of the 74 commonly altered DEGs from entorhinal cortex were screened into the DEGs PPI network, containing 32 nodes and 41 edges (Fig. 3a), and 42 of the 74 DEGs did not fall into the DEGs PPI network. As shown in Fig. 3a, the majority of the nodes in the network were down-regulated DEGs in AD samples. Among the 32 nodes, 2 central node genes were identified with the filtering of degree≥8 criteria (i.e., each node had more than 8 connections/interactions) as top 2 hub genes, which were OXT and TAC1 (Fig. 3b). Figure 3b presents these 2 genes and their first neighbor genes.

In total, 1 module (Modules 3) with score > 3 was detected by MCODE (Fig. 3c). KEGG pathway enrichment analyses showed that Module 3 consisted of 5 nodes and 10 edges, which are mainly associated with signal transduction (including multiple receptors) (Table 4).

**Table 4:** Pathway enrichment in Module 3

#Pathway ID	Pathway description	Observed gene count	False discovery rate	Matching proteins in Your network (ids)	Matching Proteins In your Network (labels)
R-HSA:416476	G alpha (q) signaling events	5	3.80E-09	ENSP00000321106, ENSP00000324270, ENSP00000332225, ENSP00000369647, ENSP00000434045	AVP,GPR68, OXTR, PMCH, TAC1
R-HSA:373076	Class A/1 (Rhodopsin-like receptors)	5	1.49E-08	ENSP00000321106, ENSP00000324270, ENSP00000332225, ENSP00000369647, ENSP00000434045	AVP,GPR68, OXTR, PMCH, TAC1
R-HSA:375276	Peptide ligand-binding receptors	4	2.80E-07	ENSP00000321106, ENSP00000324270, ENSP00000332225, ENSP00000369647	AVP,OXTR, PMCH, TAC1
R-HSA:388479	Vasopressin-like receptors	2	5.84E-06	ENSP00000324270, ENSP00000369647	AVP,OXTR

### Identification of lncRNAs and analysis of binding proteins

**Fig. 4:**
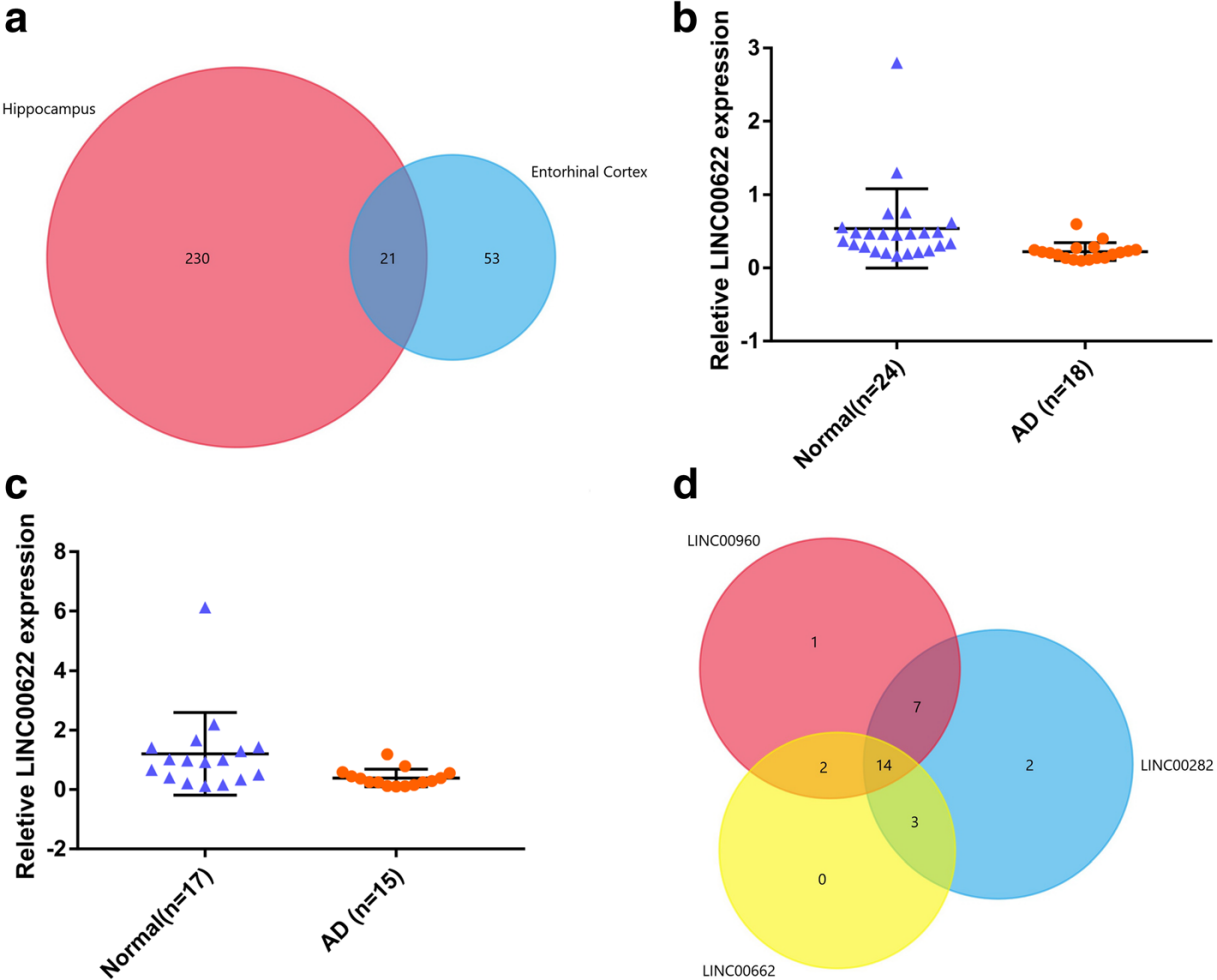
LncRNAs identification and filtration of mutual binding proteins. **a** Mutual DEGs between hippocampus and entorhinal cortex. **b** Relative expression of linc00622 in hippocampus. **c** Relative expression of linc00622 in entorhinal cortex. **d** Mutual RNA-binding proteins among linc00622, linc00282 and linc00960

21 mutual DEGs were discovered through the tool of venn by employing Funrich software, and among which 1 lncRNA, linc00622, was identified as differently expressed both in the tissues of hippocampus and entorhinal cortex between AD patients and normal controls (Fig. 4a). Figure 4b and c show the relative expressed values of linc00622 in the tissues of hippocampus and entorhinal cortex. Linc00282 and linc00960 were differently expressed in the tissues of hippocampus and entorhinal cortex, respectively. After using the online tools, University of Califorina Santa Cruz (UCSC) and the Database of RNA-binding protein specificities (RBPDB), we obtained the RNA-binding proteins lists of linc00662, linc00282 and linc00960. Then, 14 mutual RNA-binding proteins were found to be shared by these 3 lncRNAs via the tool of venn (Fig. 4d). The biofunctions of these RNA-binding proteins were summarized in the Table 5.

**Table 5:** Information of RNA-binding proteins of lncRNAs

Gene symbol	Gene title	Involved cell functions	References
EIF4B	eukaryotic initiation factor 4B	long-term plastic changes in neuron	[34]
PABPC1	cytoplasmic poly(A)-binding protein 1	inflammation	[35]
FUS	fused in sarcoma	motor neuron degeneration	[36]
Pum2	pumilio 2	neurogenesis	[37]
MBNL1	muscleblind-like protein 1	neuron development	[38]
ACO1	aconitase 1	neuron mitochondrial function	[39]
KHSRP	KH-type splicing regulatory protein	inflammation, neuron survival and growth	[40]
YTHDC1	YTH domain-containing protein 1	neuron development	[41]
SFRS9	SR-family splicing factor 9	neuronal stimulation	[42]
RBMX	RNA binding motif protein X-linked gene	neuron development	[43]
SFRS13A	SR-family splicing factor 13A	cholesterol homeostasis, AD risk	[44]
KHDRBS3	KH RNA binding domain containing, signal transduction associated 3	cell proliferation	[45]
ELAVL1	ELAV-like protein 1	neurogenesis, neuroprotection	[46, 47]
SFRS1	SR-family splicing factor 1	neuron differentiation and survival	[48, 49]

## Discussion

In general, AD is a genetically complex neurodegenerative disease and is characterized by the presence of extracellular deposition of senile plaques, intracellular NFTs and loss of neuron and synapses [50, 51]. AD affects patients’ living quality and is detrimental to their life, and further imposes a considerable burden on their families and the whole society. However, there are rare effective therapies for AD patients nowadays; it is urgent to develop novel perspectives to improve treatment outcomes [52].

We selected GSE48350 database, which contains microarray data from AD cases (aged 20–99 years) and age matched normal controls, from 4 brain regions: hippocampus, entorhinal cortex, superior frontal cortex, and post-central gyrus. Previous study has demonstrated that frontal cortical dysfunction contributed a significant extent to cognitive deficits and memory loss, which was considered as the late characteristic of AD [53]; meanwhile, AD has been widely considered as an early amnesic syndrome of hippocampal type, which on behalf of the most significant clinic feature for the diagnosis of AD [54–56], and we believed that the data between age-matched AD patients and normal controls were more convictive. Besides, entorhinal cortex is also considered as a vital brain region in characterizing AD, and aberrant changes of entorhinal cortex happen before hippocampus in the pathological mechanism of AD [57–59]. Therefore, we analyzed the data from hippocampus and entorhinal cortex tissues between aged AD cases from 69 to 99 years and age matched normal controls. We employed several types of tools to recognize critical molecular terms and mechanisms involved in AD.

We firstly used Morpheus to filtrate the DEGs from post-mortem samples between AD patients and normal controls using *p* < 0.05 and |log2FC| ≥ 1 as the criteria, and then we obtained 251 DEGs including 56 up-regulated genes and 195 down-regulated genes in hippocampus tissues, and 74 DEGs including 16 up-regulated genes and 58 down-regulated genes in entorhinal cortex tissues.

The overrepresented biological processes, cellular components and molecular functions obtained from GO analyses of DEGs from hippocampus and entorhinal cortex tissues may give valuable information about the pathogenic molecular mechanisms of AD. Among the GO terms overrepresented in AD patients, those related to memory-related processes, drug reactions, transmembrane transportation, synaptic transmission and ion homeostasis were included. These results were in accordance with previous studies that complex interrelationships of synaptic depression, cognitive impairment, aberrant drug metabolism, and imbalance of ion homeostasis existed among the nosetiology and development processes of AD [60–65]. The multiformity in the biological process of genes involved in AD indicated the complicacy of the disease. Recent studies convinced that ion channels are well-known to be involved in AD pathophysiology, especially potassium ion channel, and is emerging as a new target candidate for AD [66, 67].

Pathway enrichment analyses of DEGs from hippocampus and entorhinal cortex tissues were classified by the Reactome, KEGG and/or Wikipathway databases, respectively. The different databases provided similar information with the majority of AD-related proteins acting in 19 major pathways (13 from hippocampus and 6 from entorhinal cortex), mainly related transmembrane transportation, drug reactions, synapses function, ion homeostasis, neurogenesis and signal transduction, which were consistent with the results of GO analyses of these DEGs and previous works focused on the aetiology of AD [60–65, 68]. Besides, in the pathway analyses of 3 modules (2 for DEGs from hippocampus and 1 for entorhinal cortex), it was indicated that multiple signal transduction pathways were involved in the pathological mechanism of AD.

From the results of functional enrichment analyses of DEGs, it can be concluded that synaptic depression, cognitive impairment, aberrant drug metabolism, and imbalance of ion homeostasis participated in the pathology of AD and signaling pathways that regulate these biological phenomena would be the efficient treatment targets for AD.

Among 11 hub genes (9 from hippocampus and 2 from entorhinal cortex) obtained from PPI network analyses in this study, several genes involved in the regulation of cell survival and cell growth, such as CDC42 and VEGFA; several genes involved in the memory, learning, and cognitive functions, such as BDNF, PDYN, CALB, TH, CACNA1A, and OXT; several genes involved in the immune and neuroprotective functions, such as CD44 and TAC1. Detailed information of these genes is as seen-shown in Table 6. Specially, as far as we know, CALB, CACNA1A and OXT were identified as the hub participants in the pathological mechanism of AD for the first time in this study.

**Table 6:** Detailed information of hub genes

Hub genes	Involved cell functions	References
Hippocampus		
CDC42	actin cytoskeleton, gene expression, cell proliferation, Aβ neurotoxicity	[69–72]
VEGFA	neurogenesis, neuronal migration	[73–75]
BDNF	growth, survival and maintenance of neurons	[76, 77]
PDYN	memory, learning, and cognitive functions; drug consumption and addiction	[78–80]
CALB	long-term potentiation, synaptic plasticity, and memory functions	[81, 82]
TH	memory and recognition functions	[83, 84]
CACNA1A	learning and memory	[85, 86]
CD44	inflammation-related functions	[87–89]
TAC1	neurotrophic and inflammation related functions	[90, 91]
Entorhinal cortex		
OXT	drug addiction, anxiety and memory formation	[92–94]
TAC1	neurotrophic and inflammation related functions	[90, 91]

Given multiple biofunctions of human lncRNA, the associations between lncRNA and AD have great potential benefits to understanding the cause of AD. So far, only several lncRNAs, such as BACe1-AS [27], 51A [28], 17A [29], BC200 [95] and so on, have been validated to be involved in the pathogenesis of AD. Identifying potential diagnostic lncRNA biomarkers by employing computational methods is promising in the biomarker filtration for AD. In the present study, among 21 mutual DEGs of hippocampus and entorhinal cortex tissues, 1 lncRNA, linc00622 was identified as differently expressed both in the tissues of hippocampus and entorhinal cortex. Besides, linc00282 and linc00960 were differently expressed in the tissues of hippocampus and entorhinal cortex, respectively. 14 mutual RNA-binding proteins were proved to have close relationship with paroxysm and development of AD. Therefore, linc00622, linc00282 and linc00960 could be considered as novel potential candidates participating in the pathological mechanism of AD.

## Conclusions

Combined the results of comprehensive and systematic analyses focusing on the biological functions and interactions of the genes extracted from GSE48350 genome database of AD patients and normal controls, genes mainly related the biological functions of memory, synapse, neuron survival, drug metabolism, ion homeostasis and signal transduction were differently expressed in the hippocampus and entorhinal cortex tissues of AD patients aged from 69 to 99 years and age matched normal controls. Our study should shed some light toward a better understanding of the underlying molecular mechanisms and crucial molecular players of AD, and provide a new viewpoint for researchers with target the cause of the disease, and also these understandings need to be further validated by experiments in the future.

## Materials and methods

### Microarray data extraction and identification of DEGs

The network-based analyses of AD began with the authentication of microarray gene expression dataset. We downloaded the gene expression profile and sample information of GSE48350 from the public availability repository GEO database (https://www.ncbi.nlm.nih.gov/geo/) [96] from NCBI and European Bioinformatics Institute’s (EBI) ArrayExpress-functional genomics database (https://www.ebi.ac.uk/arrayexpress/) [97]. GSE48350 contains post-mortem brain tissue samples from diseased (patients with AD) and control (normal) conditions. Hippocampus region of brain plays significant role in memory formation, which is necessary in diagnostics since loss of memory and cognitive competence and disorientation are the early signs of AD [56]. In this study, we analyzed 18 AD samples of hippocampus region of post-mortem brain aged from 69 to 99 (mean age 84.33 ± 6.56 years) and 24 age-matched control samples of hippocampus region of post-mortem brain (mean age 82.71 ± 9.47 years), and 15 AD samples of entorhinal cortex region aged from 69 to 99 (mean age 86.47 ± 5.46 years) and 17 age-matched control samples of entorhinal cortex region (mean age 81.65 ± 9.76 years).

Morpheus (https://software.broadinstitute.org/morpheus/) [98] online tool allows researchers to carry out of GEO data to identify DEGs. A gene is defined as a DEG between the patients’ samples and the normal control samples when the *p*-value is < 0.05 and the FC is at least 2 times higher or lower (|log2FC| ≥ 1).

### Functional enrichment analyses for DEGs

Executing functional enrichment analyses for DEGs gives a functional overview of the DEGs through computing the whole conspicuousness of the gene expression. GO comprises biological process, cellular component, and molecular function, providing biological functional interpretation of large lists of genes screened from genomic studies such as microarray and proteomics experiments [99, 100]. KEGG is an encyclopedical database resource consisting of graphical diagrams of biochemical pathways for functional gene and molecules to be integrally analyzed [101, 102]. Reactome, an online bioinformatics resource of pathway information, supplies integrated analysis of the biologic reaction network [103, 104]. WikiPathway provides a database in a curated, machine readable way to analyze and visualize data [105]. Pathway analyses of KEGG, Reactome and Wikipathway were employed to illuminate how DEGs perform function through a certain path.

We selected Functional Enrichment analysis tool (Cytoscape v3.7.0), which is an autocephalous software tool employed mainly for functional enrichment and interaction network analyses of genes and proteins. Cytoscape is open source software who can integrate interaction networks of high-throughput expression data and other molecular states of genes and proteins into a unitive conceptual framework [106]. This software has been widely employed by researchers to study biological domains, the genome, proteome and metabonomics [107–110]. The functional enrichment analyses for the up-regulated and down-regulated DEGs and pathways were performed via Cytoscape and its plugins, ClueGO v2.5.3 and Cluepedia v1.5.3, using *p* < 0.05 as the selected criterion in the present study.

### Construction of PPI network for DEGs and recognition of hub proteins

PPI was employed to analyze the interrelationship among DEGs, and further illustrate the models of genes which play significant roles in physiological and pathological status. The STRING database (https://www.string-db.org/) [111] supplies information about the predicted and experimental interrelationships of proteins, and helps to assess and integrate PPI, including direct (physical) and indirect (functional) correlations [112, 113]. In this study, the DEGs were mapped into PPI using STRING database v10.5. Then, Cytoscape software was employed to visualize the PPI network. The network module was one of the peculiarities of the protein network and contains peculiar biological importance. The MCODE (v1.5.1) was employed to identify remarkable modules in this PPI network. Degree cutoff = 2, Node Score Cutoff = 0.2, and K-Core = 2 were set as the advanced settings. MCODE was applied to filtrate hub proteins within the PPI network. At last, the enrichment analyses of the DEGs in different modules were also conducted by the STRING database.

### Identification of lncRNAs and binding proteins prediction

Mutual DEGs were discovered through the tool of venn by employing Funrich (3.1.3) software. The relative expressed values of linc00622 in the tissues of hippocampus and entorhinal cortex were analyzed by Graphpad Prism (7.0) software. Online tools, UCSC (https://genome.ucsc.edu/index.html) [114, 115] and RBPDB (http://rbpdb.ccbr.utoronto.ca/index.php) [116, 117] were used to obtain the RNA-binding proteins lists of linc00662, linc00282 and linc00960. Then, mutual RNA-binding proteins shared by these 3 lncRNAs were found via the tool of venn.

## Additional files

Additional file 1 **Table S1.** Information for samples in the included datasets. (ArrayExpress datasets). Included detailed information of samples of hippocampus and entorhinal cortex regions extracted from GSE48350. (XLSX 14 kb)

Additional file 2 **Table S2.** Information for the DEGs identified from the GEO dataset (|log2FC| ≥ 1, *p* value< 0.05). Included detailed information of all DEGs screened from hippocampus and entorhinal cortex regions. (XLSX 31 kb)

Additional file 3 **Table S3.** Information for biological process analysis of DEGs from hippocampus. Included detailed information of results of biological process analysis of DEGs from hippocampus. (XLSX 32 kb)

Additional file 4 **Table S4.** Information for cellular component analysis of DEGs from hippocampus. Included detailed information of results of cellular component analysis of DEGs from hippocampus. (XLSX 21 kb)

Additional file 5 **Table S5.** Information for molecular function analysis of DEGs from hippocampus. Included detailed information of results of molecular function analysis of DEGs from hippocampus. (XLSX 17 kb)

Additional file 6 **Table S6.** Information for biological process analysis of DEGs from entorhinal cortex. Included detailed information of results of biological process analysis of DEGs from entorhinal cortex. (XLSX 16 kb)

Additional file 7 **Table S7.** Pathway enrichment analyses of DEGs. Included detailed information of pathway enrichment analyses of DEGs from hippocampus and entorhinal cortex. (XLSX 12 kb)

## Data Availability

All data generated or analyzed during this study are included in this published article [and its supplementary information files].

## Abbreviations

AD: Alzheimer’s disease
APP: amyloid precursor protein
Aβ: amyloid-β
DEGs: differentially expressed genes
EBI: European Bioinformatics Institute
FC: fold change
GEO: Gene Expression Omnibus
GO: Gene Ontology
KEGG: Kyoto Encyclopedia of Genes and Genomes
LncRNA: long non-coding RNA
MCODE: Molecular Complex Detection
NCBI: National Center of Biotechnology Information
NFTs: neurofibrillary tangles
PPI: protein-protein interaction
PSEN1: presenilin 1
PSEN2: presenilin 2
RBPDB: the Database of RNA-binding protein specificities
STRING: Search Tool for the Retrieval of Interacting Genes
UCSC: University of California Santa Cruz
